# Psychological Traumas and Cardiovascular Disease: A Case-Control Study

**DOI:** 10.3390/healthcare9070875

**Published:** 2021-07-12

**Authors:** Federica Galli, Carlo Lai, Teresa Gregorini, Chiara Ciacchella, Stefano Carugo

**Affiliations:** 1 Department of Dynamic and Clinical Psychology, and Health Studies, Sapienza Università di Roma, 00185 Rome, Italy; carlo.lai@uniroma1.it (C.L.); chiara.ciacchella@uniroma1.it (C.C.); 2 UOC Cardiology, ASST SS. Paolo e Carlo, S. Paolo Hospital, 20147 Milan, Italy; teresa.gregorini01@universitadipavia.it; 3 Department of Clinical Sciences and Community Health, University of Milan, 20122 Milan, Italy; stefano.carugo@unimi.it; 4 UOC Cardiology, Fondazione IRCCS Ca’ Granda Ospedale Maggiore Policlinico, 20122 Milan, Italy

**Keywords:** cardiovascular diseases, adverse childhood experiences, trauma, women

## Abstract

Adverse childhood experiences could be important determinants of adult disease. The present study analyzed the association between early traumatic experiences and the onset of cardiovascular disease (CVDs). It was hypothesized that patients with CVD would report a higher number of traumatic experiences during childhood and that this association would be stronger in women. The Traumatic Experiences Checklist (TEC) was fulfilled by 75 patients with a first-time diagnosis of CVD and 84 healthy controls randomly selected from the general population. The two groups were not balanced for age and sex. Multivariate analyses of covariance (MANCOVAs) and analyses of covariance (ANCOVAs), with group (clinical vs. control) and gender (male vs. female) as between-subjects factors, and age of participants as covariate, were performed on the number and the impact of the traumatic experiences (emotional neglect, emotional abuse, physical abuse, sexual harassment, and sexual abuse) for the three age group in which the trauma was experienced (from 0 to 10, from 11 to 18, from 19 years onwards). The main results showed that participants with CVDs have experienced a higher number of early traumatic experiences compared to the control group, such as emotional neglect (*p* = 0.023), emotional abuse (0.008 ≤ *p* ≤ 0.033), and physical abuse (0.001 < *p* ≤ 0.038). The results also revealed that women with CVDs have experienced more traumatic events compared to the women of the control group (0.001 < *p* ≤ 0.020). These results seem to highlight an association between traumatic experiences in childhood and CVD in adulthood, particularly in women. Such findings could have relevant implications for clinical practice, suggesting the importance of adopting an integrated approach in the care of the patient with cardiovascular diseases paying attention also to the clinical psychological risk factors.

## 1. Introduction

Cardiovascular diseases (CVDs) are the primary cause of death in the world [1]. According to the European cardiovascular disease statistics of 2017 [2], cardiovascular diseases (CVDs) are responsible for the death of around 3.9 million people *per* year, which is roughly 45% of overall deaths in Europe. These pieces of evidence stressed the importance of primary prevention, identifying the risk factors in the development of CVDs. European guidelines on cardiovascular disease prevention in clinical practice [3] underline the importance of several factors, such as smoking, gender, age, familiarity, hypertension, dyslipidemia, diabetes, and some inflammatory biomarkers (e.g., C-reactive protein). However, risk factors are not exclusively biological and/or behavioral: certain psychological variables, like depression, anxiety, hostility, social isolation, and mental stress conditions, have been recognized as playing a role in the development of CVDs and worsening the progression [3,4,5,6]. However, the important scientific statement recently published by the American Heart Association [6] did not yet consider the potential role of early traumas as a possible risk factor for CVDs.

Traumatic childhood experiences are well known to increase the risk of developing mental illnesses [7], as well as the spread of the onset of physical illnesses, such as gastrointestinal [8], cancer [7], autoimmune [9], and dermatological diseases [10]. In this framework, there is sparse evidence on the importance of taking into consideration the role of adversities or trauma on the development of CVDs [4,6,11].

Felitti et al. [12] showed that childhood adversities such as negligence or family conflict were associated with CVD in adulthood, and that this association was directly proportional to the number of adverse experiences lived. Other longitudinal studies found that individuals with adverse experiences or trauma during childhood had a higher risk of developing CVD [13,14]. The adverse childhood experiences (ACE) study suggested highly significant associations between ACE and ischemic cardiovascular disease, also underlining how these psychological factors were statistically more relevant than traditional risk factors [15].

The age of the person at trauma has been pointed out as another predictive factor of cardiovascular diseases [16,17]. Furthermore, different types of traumatic experiences seemed to be associated with CVDs [14,18,19,20,21], especially in women [11,22]. Recently, a longitudinal study showed how childhood adversities and trauma are associated with CVD in adulthood, pointing out that subjects who had experienced more adverse experiences during childhood had a 50% higher risk of CVD incidence compared to those who reported few or no stressful experiences [23]. A large population-based multi-ethnic urban cohort study tested whether child maltreatment, such as emotional neglect, emotional abuse, physical abuse, and sexual abuse, was associated with a higher risk of self-reported history of CVD. The study confirmed that child maltreatment is significantly associated with a higher risk of CVD later in life, and that this association remained significant after adjusting for potentially relevant covariates [24].

However, to the best of our knowledge, no previous studies have investigated the role of traumatic experiences by means of a case-control design, comparing patients with heart disease and healthy people. This is also the first study adopting a psychodiagnostic tool, describing in detail the type and age of trauma.

The aim of the present study was to verify the association between traumatic experiences and the onset of CVDs. The main hypothesis was that the patients with CVDs would report a higher number of traumatic experiences, especially at an early age, compared to the healthy control. 

## 2. Materials and Methods

### 2.1. Sample

Participants were recruited from November 2017 to July 2019. The patients had a first-time diagnosis of CVD requiring hospitalization. The study had two populations: the clinical group consisted of consecutive patients with a cardiovascular disease who entered the Cardiology Unit of the San Paolo Hospital in Milan. The diagnosis and clinical workouts were carried out by the cardiologists of the Cardiology Unit of the San Paolo hospital (Milan). The control group was recruited by the general population through online announcements and consisted of individuals without recent or lifetime diagnosis of CVDs and a metabolic syndrome. Participants of both groups were excluded if they were older than 75 years, were unable to understand Italian, or had psychosis, mental retardation, intellectual disability, and/or cognitive delay (the assessment by psychological tests was preceded by a clinical interview).

The study was conducted according to the guidelines of the Declaration of Helsinki and approved by the Ethical Committee of the Santi Paolo e Carlo Hospital, Milan (Italy) (Milano Area 1).

An a priori power analysis using the results of a previous study [25] was performed. The required sample size was of 48 participants for each group (power = 80%; *p*-value = 0.05).

### 2.2. Assessment of Traumatic Experiences

The Traumatic Experience Checklist (TEC) [26] is a self-report questionnaire designed to screen potentially traumatizing events. The TEC assesses the presence of 29 types of traumatic experiences, which are organized in five subscales: emotional neglect (e.g., being left alone, lack of affection), emotional abuse (e.g., being belittled, made fun of, insulted, verbally threatened or unfairly punished), physical abuse (e.g., being affected, tortured or injured), sexual harassment (acts of a sexual nature that do not involve physical contact) and sexual abuse (unwanted sexual acts that involve physical contact). The questionnaire elicits information about the number of traumas experienced by the participant and the impact of each traumatic experience, ranging from 1 (*none*) to 5 (*an extreme amount*). Moreover, the questionnaire elicits information about how old the participant was when he/she experienced each event. This allows the researchers to organize the traumatic experiences in 3 different age groups (from 0 to 10, from 11 to 18 and from 19 years onward).

### 2.3. Experimental Procedure

After signing the informed consent, the TEC was administered to each participant by an expert psychologist (T.G.). For the clinical group, the data related to the diagnosis were collected by cardiologists from the patient’s medical records.

### 2.4. Statistical Analyses

To verify the reliability of measurements, the Kolmogorov–Smirnov normality test was conducted on the data of the TEC related to the number of traumatic experiences, reporting a *p*-value lower than 0.01. In order to normalize the data sample distribution of the number of traumas (x), a power transformation (logarithmic transformation) was performed [Y = log10 (x + 1)].

In order to capture the differences in traumatic experiences between the clinical and the control groups, the following analyses were performed. 

The 2 × 2 multivariate analysis of variance (MANOVA), with group (clinical vs. control) and gender (male vs. female) as between-subjects factors, was performed on the number of traumatic experiences (emotional neglect, emotional abuse, physical abuse, sexual harassment, and sexual abuse subscales) in the different age groups (from 0 to 10, from 11 to 18, from 19 years onwards, and total). Moreover, 2 × 2 analyses of variance (ANOVAs), with group (clinical vs. control) and gender (male vs. female) as between-subjects factors, were performed using the number of each form of traumatic experience (emotional neglect, emotional abuse, physical abuse, sexual harassment, and sexual abuse subscales) in each age group (from 0 to 10, from 11 to 18, from 19 years onward, and in total). Planned comparisons were carried out, using the least significant difference (LSD) test for the planned comparisons related to the “group” effect, and the unequal N HSD test for the planned comparisons related to the “gender” and “group *per* gender” effects.

The 2 × 2 MANOVA, with group (clinical vs. control) and gender (male vs. female) as between-subjects factors, was performed on the impact of the traumatic experiences (emotional neglect, emotional abuse, physical abuse, sexual harassment, and sexual abuse subscales) in the different age groups (from 0 to 10, from 11 to 18, from 19 years onward). Moreover, 2 × 2 ANOVAs, with group (clinical vs. control) and gender (male vs. female) as between-subjects factors, were performed on the impact of each traumatic experience (emotional neglect, emotional abuse, physical abuse, sexual harassment, and sexual abuse subscales) in each age group (from 0 to 10, from 11 to 18, from 19 years onward). Planned comparisons were carried out, using the least significant difference (LSD) test for the planned comparisons related to the “group” effect, and the unequal N HSD test for the planned comparisons related to the “gender” and “group *per* gender” effects.

All statistical analyses were performed using Statistica 8 software (StatSoft, Inc., Tulsa, OK, USA, 2007).

## 3. Results

The final sample was composed of 159 participants. The clinical group consisted of 75 patients (mean age (m.a.) 54.89 ± 14.49 years), 55 men (m.a. 51.67 ± 14.689 years) and 20 women (m.a. 63.75 ± 9.63 years). Table 1 reported the detailed diagnoses of the clinical group. The control group consisted of 84 participants (m.a. 49.69 ± 5.67), 36 men (m.a. 51.17 ± 6.49 years) and 48 women (m.a. 48.58 ± 4.75 years).

The 2 × 2 MANCOVA group (clinical vs. control) *per* gender (male vs. female), with age of participants as covariate, performed on the number of the traumatic experiences (emotional neglect, emotional abuse, physical abuse, sexual harassment, and sexual abuse subscales) in the different age groups (from 0 to 10, from 11 to 18, from 19 years onwards, and total) showed a significant main effect of group [Wilks’ Lambda = 0.68; F (16,139) = 3.99; *p* < 0.001], a significant main effect of gender [Wilks’ Lambda = 0.83; F (16,139) = 1.75; *p* = 0.044], a significant interaction effect group *per* gender [Wilks’ Lambda = 0.82; F (16,139) = 1.88; *p* = 0.027], and a significant main effect of age [Wilks’ Lambda = 0.81; F (16,139) = 2.09; *p* = 0.012].

The 2 × 2 ANCOVAs group (clinical vs. control) *per* gender (male vs. female), with age of participants as covariate, performed on the number of each traumatic experience (emotional neglect, emotional abuse, physical abuse, sexual harassment, and sexual abuse subscales) in each age group (from 0 to 10, from 11 to 18, from 19 years onwards, and total) showed the following results (Table 2). A significant main effect of group was found on the emotional neglect (0–10), emotional abuse (0–10, 11–18, and total), physical abuse (0–10, 11–18, +19, and total), sexual harassment (+19 and total), and sexual abuse (total) subscales. A significant main effect of gender was found on the emotional abuse (0–10 and total), physical abuse (+19 and total), and sexual harassment (+19 and total) subscales. A significant interaction effect group *per* gender was found on the emotional abuse (0–10), physical abuse (+19 and total), and sexual harassment (+19 and total) subscales. A significant main effect of age was found on the emotional neglect (+19), emotional abuse (11-18 and total), physical abuse (+19), and sexual harassment (+19) subscales.

From the planned comparisons emerged that the clinical group reported a higher number of traumatic experiences compared to the control group on the emotional neglect (0–10), emotional abuse (0–10, 11–18, and total), and physical abuse (0–10, 11–18, and total) subscales (see Table 2). Moreover, the men of the clinical group showed a lower number of traumatic experiences compared to women of the clinical group on the sexual harassment (total) subscale, and a higher number of traumatic experiences compared to the men of the control group on the physical abuse (total) subscale (Table 2). Finally, women in the clinical group showed a higher number of traumatic experiences compared to those of the control group on the emotional abuse (0–10), physical abuse (0–10 and total), and sexual harassment (total) subscales (Table 2). 

The 2 × 2 MANCOVA group (clinical vs. control) *per* gender (male vs. female), with age of participants as covariate, performed on the impact of the traumatic experiences (emotional neglect, emotional abuse, physical abuse, sexual harassment, and sexual abuse subscales) in the different age groups (from 0 to 10, from 11 to 18, from 19 years onwards) showed a significant main effect of group [Wilks’ Lambda = 0.69; F (13,142) = 4.91; *p* < 0.001], a significant main effect of gender [Wilks’ Lambda = 0.85; F (13,142) = 1.86; *p* = 0.039], a significant interaction effect group *per* gender [Wilks’ Lambda = 0.85; F (13,142) = 1.98; *p* = 0.026], and a significant main effect of age [Wilks’ Lambda = 0.85; F (13,142) = 1.95; *p* = 0.029]. 

The 2 × 2 ANCOVAs group (clinical vs. control) *per* gender (male vs. female), with age of participants as covariate, performed on the impact of each traumatic experience (emotional neglect, emotional abuse, physical abuse, sexual harassment, and sexual abuse subscales) in each age group (from 0 to 10, from 11 to 18, from 19 years onwards) showed the following results (Table 3). A significant main effect of group was found on the emotional neglect (0–10), emotional abuse (0–10 and 11–18), and physical abuse (0–10, 11–18, and +19), and sexual harassment (+19) subscales. A significant main effect of gender was found on the emotional abuse (0–10) and sexual harassment (+19) subscales. A significant interaction effect group *per* gender was found on the emotional abuse (0–10) and sexual harassment (+19) subscales. A significant main effect of age was found on the emotional neglect (+19), emotional abuse (0–10 and 11–18), physical abuse (+19), and sexual harassment (+19) subscales.

From the planned comparisons emerged that the clinical group reported higher impact scores compared to the control group on the emotional neglect (0–10), emotional abuse (0–10 and 11–18), and physical abuse (0–10) subscales (Table 3). Finally, women in the clinical group reported higher impact scores than those in the control group on emotional abuse (0–10), and physical abuse (0–10) subscales (Table 3).

## 4. Discussion

In line with our main hypothesis, patients with CVDs reported a greater number of traumas in the first 10 years of life compared to healthy controls. These findings definitively confirmed the role of adverse events experienced in childhood in increasing the risk of cardiovascular diseases, as previously suggested [27,28].

The clinical group reported a higher number and greater impact of emotional neglect in the first 10 years of life compared to the control. Specifically, experiencing emotional neglect, emotional abuse, physical abuse, starting from early childhood, seemed to be associated with the presence of a CVD. Moreover, the amount and the impact of emotional abuse experienced during childhood and adolescence were higher in the patients with the CVDs compared to the control group, further supporting the role of traumatic events in the development of a CVD [24].

Likewise, the participants of the clinical group reported a greater number and higher impact of physical abuse, especially during childhood and adolescence, compared to the participants of the control group. This result further supports the strong association between episodes of physical violence in early life and the risk of CVDs [29] and other subclinical conditions, as in the case of a typical nocturnal drop in blood pressure, the increase in diastolic pressure and heart rate [19,30]. Cardiovascular health also seems to be affected by physical abuse by indirect pathways: violence is often associated with depression, aggression, the use of substances or reduced physical activity, all important risk factors for CVDs [29,31].

Furthermore, our results showed a greater number of experiences of harassment and sexual abuse in the clinical group. Sexual abuse has been associated with a greater risk of CVD [16,32] and some subclinical CVD markers, such as a greater thickness of the intimate carotid artery [21].

According to our hypothesis, the women of the clinical group reported more traumatic experiences, such as emotional abuse, physical abuse and sexual harassment, compared to the men of the same group and to the women of the control group. These results are consistent with other findings, showing important implications for understanding sex differences in CVD risk [11,33]. According to the recent literature, stress could have a greater impact on women’s cardiovascular health compared to men’s, who could be less vulnerable to those effects [34,35]. Adverse childhood experiences could be considered as independent risk factors for the incidence of ischemic cardiomyopathies in women [22,28]. Moreover, it was found that in women, the presence of childhood traumatic experiences was associated with specific risk factors for CVD, such as an increase in inflammatory and immune activity [36] and high levels of the protein C-reactive [37], decreased heart rate variability [20] and an increased neuroendocrine stress reactivity [38].

This association between early traumatic experiences and CVD could be explained by considering three different points of view: behavioral factors, mental pathology and biological mechanisms. As regards the behavioral factors, several studies [6,12,39,40] highlighted the association between behaviors harmful to health, such as smoking, excessive alcohol use, overeating and weight gain, that increase the risk of cardiovascular disease, and trauma in childhood.

Subsequently, another important mediator between trauma and cardiovascular pathologies could be the presence of mental pathology. In particular, the relationship between an adverse childhood experience (ACE) and the development of a mental disorder [7], such as post-traumatic stress disorder, and mood and anxiety disorders, seemed to increase the risk of the onset of CVD [5].

The biological processes potentially involved in mediating the relationship between childhood trauma and cardiovascular disease are different and complex.

In the event of exposure to a stressor, there is an activation of the sympathetic nervous system (SNS) which, in turn, triggers inflammation and activation of the immune system [41]. Therefore, the cardiovascular outcome could be mediated by an alteration of the autonomic nervous system, in terms of increased sympathetic tone or reduced parasympathetic tone [6,42]. It would seem that, in case of trauma, there is a greater response of the SNS to a stressor in adulthood, consequently increasing the levels of circulating catecholamines [43]. Catecholamines can cause lesions to the endothelium of the coronary arteries, and promote the development of atherosclerosis [44], a process that can determine the onset of a CVD [45]. The autonomic nervous system dysregulation observed in subjects who had traumatic experiences [42] can cause a reduction in heart rate variability [46], considered a precursor of cardiovascular diseases [47].

To explain the association between CVD and traumatic experiences, the role of the hypothalamic-pituitary-adrenal (HPA) axis must also be taken into consideration: some studies show an alteration of the HPA axis in adults who have experienced traumatic childhood experiences [38], characterized by less cortisol secretion and flatter daytime oscillations [48].

This alteration in circulating cortisol levels has consequences on the cardiovascular system: it has been shown that the lower the fluctuation of cortisol levels within 24 h, the greater the risk of coronary artery calcification [37].

Moreover, the evidence underlined the role of inflammation in cardiovascular disease, showing that patients with high levels of markers associated with the inflammatory response report an increased risk of developing diabetes and cardiovascular pathologies [6,49].

Several studies highlighted a strong relationship between childhood adversity and an alteration in the inflammatory response [50], finding an increase in the levels of anti-inflammatory cytokines and, therefore, of inflammatory activity [51]. It would seem that an increase in the levels of inflammatory cytokines, such as interleukin 6, the Creactive protein and the tumor necrosis factor a, compromise endothelial functioning, promoting the process of atherosclerosis [52].

Lastly, several reviews described the association between ACE and DNA methylation [53], and between it and cardiovascular disease [54], thus opening the way to a new etiology hypothesis capable of explaining the association between ACE and CVD. Accordingly, Misiak et al. [55] showed that emotional abuse was associated with the hypomethylation of LINE-1 in DNA, a reduction associated with the risk of ischemic heart disease and stroke [56]. Anomalies concerning the NR3C1 gene [57], implicated in the atherosclerosis process and associated with heart disease [58], were observed in adult victims of abuse and child abuse. In addition, an up-regulation of the genes involved in the inflammatory activity and a down-regulation of the genes involved in glucocorticoid signaling were noted [59].

Therefore, the greater activation of cytokines, the dysregulation of the immune system, and the dysregulation of the SNS and the HPA axis, consequent to the exposure to a traumatic experience in the first years of life, seem to determine a series of effects in a waterfall that will affect the cardiovascular system, increasing the risk of disease onset.

### Limitations and Directions for Future Research

The self-report tools, despite the adequate psychometric properties, suffer from some prejudices such as, for example, social desirability and recall bias, which could lead to a falsification of the answer. In future research, therefore, forms of multi-method evaluation can be examined, alongside this type of tool, such as, for example, interviews. Moreover, the data should be interpreted with caution due to the limited sample size and the gender discrepancy between the two groups, with a greater percentage of women in the control group and, on the contrary, of men in the experimental group. Furthermore, despite the age of the participants was taken into account in the analyses as covariate, the discrepancy between the two groups should be considered as a limit. In particular, the mean age of the clinical group is not only older but there is also a wider age range than the control group.

Thus, new studies with balanced samples and a greater number of participants will be required. Moreover, further studies are needed to examine the role of the duration of traumatic events and the possible mechanisms that mediate the link between trauma and cardiovascular diseases, considering also the role of other risk factors.

Despite these limitations, the study presented some important strengths, such as structuring a research design between groups, and the use of a well-validated tool like TEC. Another important strength of the study was the use of patient medical records to obtain reliable information about the diagnosis and the health status of the whole group of participants. Furthermore, TEC allowed evaluating different aspects of trauma, such as type, age and impact.

## 5. Conclusions

The rationale behind the study seemed to be confirmed by the statistically significant results, thus confirming an association between the presence of childhood traumatic experiences and an increased risk of developing cardiovascular disease in adulthood. Specifically, the types of traumas mostly associated with CVDs were emotional neglect, emotional and physical abuse, harassment and sexual abuse, especially if experienced before 18 years of age. The study confirmed that childhood trauma has a greater impact on adult cardiovascular health, and that the burden is worst in women. This evidence, together with the substantial literature on the matter, should push to promote preventive and timely intervention activities, especially with children and adolescent victims of abuse and mistreatment. Furthermore, this evidence should be translated into clinical practice to promote an integrated approach, especially in taking care of a patient with cardiovascular disease. If it is true that traumas, as well as other psychological and behavioral factors [60], can be considered risk factors for the onset of the same, it is also true that they may have an important weight in determining the outcome of the rehabilitation process, outlining the involvement of clinical psychologists in a multidisciplinary team that is able to treat the patient as a whole [6].

## Figures and Tables

**Table 1:** ICD-10 diagnoses (main category and sub-categories) of cardiovascular diseases in the clinical group (*n* = 75).

ICD-10 Code	*N* (%)
I20-I25 Ischemic heart disease	42 (56)
I20 Angina pectoris	2 (2.67)
I21 Acute myocardial infarction	25 (33.33)
I24 Other acute ischemic heart diseases	15 (20)
I30-I52 Other forms of heart disease	29 (38.67)
I30 Acute pericarditis	4 (5.33)
I42 Cardiomyopathy	3 (4)
I44 Atrioventricular and left bundle branch block	2 (2.67)
I48 Atrial fibrillation and flutter	12 (16)
I50 Heart failure	8 (10.67)
I26-I28 Pulmonary heart disease and diseases of pulmonary circulation	4 (5.33)
I26 Pulmonary embolism	4 (5.33)

Note. ICD–10 = International Classification of Diseases, tenth revision.

**Table 2:** The 2 × 2 analyses of covariance (ANCOVAs), with group [clinical (CLN) vs. control (CNT)] and gender [male (m) vs. female (f)] as between-subjects factors, and age of participants as covariate, performed on the number of each traumatic experience (emotional neglect, emotional abuse, physical abuse, sexual harassment, and sexual abuse subscales) in each age group (from 0 to 10, from 11 to 18, from 19 years onwards, and total) (*n* = 159).

Traumatic Experiences	Clinical Group (*n* = 75)		Control Group (*n* = 84)		Group		Gender		Group × Gender		Age		
	Male (*n* = 55)	Female (*n* = 20)	Male (*n* = 36)	Female (*n* = 48)									
	M (SD)	M (SD)	M (SD)	M (SD)	F (1154)	*p*	F (1154)	*p*	F (1154)	*p*	F (1154)	*p*	Planned Comparison
Emotional neglect													
0–10	0.18 (0.55)	0.35 (0.59)	0.06 (0.33)	0.08 (0.28)	7.34	**0.007**	2.88	0.092	1.21	0.272	0.12	0.727	CLN > CNT *p* = 0.023
11–18	0.04 (0.19)	0.00 (0.00)	0.00 (0.00)	0.13 (0.49)	0.55	0.460	0.73	0.395	2.67	0.104	0.01	0.916	
+19	0.07 (0.26)	0.00 (0.00)	0.05 (0.23)	0.10 (0.47)	0.22	0.641	0.01	0.922	0.02	0.865	8.52	**0.004**	
Total	0.29 (0.63)	0.35 (0.59)	0.11 (0.40)	0.31 (0.72)	3.67	0.057	2.94	0.088	<0.01	0.966	3.06	0.082	
Emotional abuse													
0–10	0.13 (0.34)	0.40 (0.50)	0.11 (0.52)	0.08 (0.35)	12.27	**0.001**	6.21	**0.014**	7.67	**0.006**	3.28	0.072	CLN > CNT *p* = 0.033; fCLN > fCNT *p* = 0.020
11–18	0.14 (0.36)	0.05 (0.22)	0.00 (0.00)	0.04 (0.20)	7.25	**0.008**	0.01	0.913	0.53	0.466	6.62	**0.011**	CLN > CNT *p* = 0.015
+19	0.00 (0.00)	0.00 (0.00)	0.00 (0.00)	0.04 (0.20)	0.67	0.413	1.36	0.245	0.76	0.377	0.22	0.636	
Total	0.27 (0.52)	0.45 (0.51)	0.11 (0.52)	0.16 (0.43)	16.36	**<0.001**	5.57	**0.019**	2.74	0.099	7.86	**0.006**	CLN > CNT *p* = 0.008
Physical abuse													
0–10	0.20 (0.52)	0.35 (0.59)	0.00 (0.00)	0.00 (0.00)	19.44	**<0.001**	1.88	0.173	1.82	0.179	0.04	0.847	CLN > CNT *p* < 0.001; fCLN > fCNT *p* = 0.006
11–18	0.07 (0.26)	0.10 (0.31)	0.00 (0.00)	0.02 (0.14)	4.73	**0.031**	0.58	0.445	0.05	0.816	0.21	0.646	CLN > CNT *p* = 0.038
+19	0.02 (0.13)	0.10 (0.31)	0.00 (0.00)	0.00 (0.00)	11.15	**<0.001**	5.08	**0.026**	6.11	**0.014**	5.61	**0.019**	
Total	0.29 (0.60)	0.55 (0.69)	0.00 (0.00)	0.02 (0.14)	34.71	**<0.001**	5.12	**0.025**	4.07	**0.045**	1.27	0.261	CLN > CNT *p* < 0.001; mCLN > mCNT *p* = 0.013; fCLN > fCNT *p* < 0.001
Sexual harassment													
11–18	0.00 (0.00)	0.05 (0.22)	0.00 (0.00)	0.00 (0.00)	2.48	0.117	3.08	0.081	2.74	0.100	0.13	0.723	
+19	0.00 (0.00)	0.05 (0.22)	0.00 (0.00)	0.00 (0.00)	6.75	**0.010**	5.25	**0.023**	6.20	**0.014**	4.79	**0.030**	
Total	0.00 (0.00)	0.10 (0.31)	0.00 (0.00)	0.00 (0.00)	9.12	**0.002**	8.59	**0.004**	8.99	**0.003**	1.73	0.190	mCLN < fCLN *p* = 0.017; fCLN > fCNT *p* = 0.017
Sexual abuse													
0–10	0.00 (0.00)	0.05 (0.22)	0.00 (0.00)	0.00 (0.00)	1.43	0.233	2.45	0.120	1.82	0.179	1.81	0.180	
+19	0.02 (0.13)	0.05 (0.22)	0.00 (0.00)	0.00 (0.00)	3.74	0.055	0.93	0.336	1.05	0.306	0.56	0.456	
Total	0.02 (0.13)	0.10 (0.31)	0.00 (0.00)	0.00 (0.00)	5.31	**0.022**	2.93	0.089	2.68	0.103	0.03	0.872	

Note. M = mean; SD = standard deviation; F = Fisher; *p* = *p* value; **bold** = ***p* < 0.05;** the F and *p* values were calculated on the transformed scores (Y) of the number of traumatic experiences (x) [Y = log_10_ (x + 1)].

**Table 3:** The 2 × 2 analyses of covariance (ANCOVAs) with group [clinical (CLN) vs. control (CNT)] and gender [male (m) vs. female (f)] as between–subjects factors, and age of participants as covariate, performed on the impact of each traumatic experience (emotional neglect, emotional abuse, physical abuse, sexual harassment, and sexual abuse subscales) in each age group (from 0 to 10, from 11 to 18, from 19 years onwards) (*n* = 159).

Impact	Clinical Group (*n* = 75)		Control Group (*n* = 84)		Group		Gender		Group × Gender		Age		
	Male (*n* = 55)	Female (*n* = 20)	Male (*n* = 36)	Female (*n* = 48)									
	M (SD)	M (SD)	M (SD)	M (SD)	F (1154)	*p*	F (1154)	*p*	F (1154)	*p*	F (1154)	*p*	Planned Comparison
Emotional neglect													
0–10	0.48 (1.36)	1.05 (1.82)	0.12 (0.75)	0.31 (1.09)	7.03	**0.009**	3.56	0.061	1.16	0.282	0.51	0.475	CLN > CNT *p* = 0.044
11–18	0.11 (0.57)	0.00 (0.00)	0.00 (0.00)	0.42 (1.40)	0.72	0.396	1.25	0.265	2.64	0.106	0.12	0.733	
+19	0.18 (0.70)	0.00 (0.00)	0.14 (0.68)	0.25 (1.00)	0.04	0.848	0.04	0.834	0.106	0.744	5.71	**0.018**	
Emotional abuse													
0–10	0.36 (0.99)	1.20 (1.82)	0.22 (0.96)	0.23 (0.93)	13.05	**<0.001**	7.09	**0.009**	7.91	**0.005**	4.87	**0.029**	CLN > CNT *p* = 0.039; fCLN > fCNT *p* = 0.025
11–18	0.44 (1.19)	0.25 (1.12)	0.00 (0.00)	0.17 (0.83)	6.45	**0.012**	0.21	0.649	0.06	0.799	6.62	**0.011**	CLN > CNT *p* = 0.046
+19	0.00 (0.00)	0.00 (0.00)	0.00 (0.00)	0.17 (0.83)	0.76	0.383	1.18	0.278	0.85	0.357	0.06	0.799	
Physical abuse													
0–10	0.56 (1.34)	1.22 (1.95)	0.00 (0.00)	0.00 (0.00)	20.89	**<0.001**	3.23	0.074	3.01	0.085	<0.01	0.970	CLN > CNT *p* < 0.001; fCLN > fCNT *p* = 0.001
11–18	0.22 (0.89)	0.50 (1.54)	0.00 (0.00)	0.10 (0.72)	4.06	**0.046**	1.78	0.184	0.41	0.521	0.04	0.832	
+19	0.09 (0.67)	0.25 (0.79)	0.00 (0.00)	0.00 (0.00)	7.53	**0.007**	1.90	0.171	2.53	0.113	4.44	**0.037**	
Sexual harassment													
11–18	0.00 (0.00)	0.25 (1.12)	0.00 (0.00)	0.00 (0.00)	2.48	0.117	3.08	0.081	2.74	0.100	0.13	0.723	
+19	0.00 (0.00)	0.20 (0.89)	0.00 (0.00)	0.00 (0.00)	6.75	**0.010**	5.26	**0.023**	6.19	**0.014**	4.79	**0.030**	
Sexual abuse													
0–10	0.00 (0.00)	0.25 (1.12)	0.00 (0.00)	0.00 (0.00)	1.43	0.233	2.45	0.120	1.82	0.179	1.81	0.180	
+19	0.09 (0.67)	0.25 (1.12)	0.00 (0.00)	0.00 (0.00)	3.74	0.055	0.93	0.336	1.05	0.306	0.56	0.456	

Note. M = mean; SD = standard deviation; F = Fisher; *p* = *p* value; **bold** = ***p* < 0.05**.

## Data Availability

The data presented in this study are available on justified request from the corresponding author. The data are not publicly available due to privacy and ethical reasons.
